# Comparison of penetrating keratoplasty outcomes with or without microwave thermokeratoplasty

**DOI:** 10.1038/s41598-021-85470-5

**Published:** 2021-03-16

**Authors:** Osamu Hieda, Koichi Wakimasu, Shigeru Kinoshita, Chie Sotozono

**Affiliations:** 1 Department of Ophthalmology, Kyoto Prefectural University of Medicine, 465 Kajii-cho, Hirokoji-agaru, Kawaramachi-dori, Kamigyo-ku, Kyoto, 602-0841 Japan; 2 Baptist Eye Institute, Kyoto, Japan

**Keywords:** Diseases, Medical research, Optics and photonics

## Abstract

Microwave thermokeratoplasty (MTK) is a surgical procedure for the correction of pathologic corneal steepening. The purpose of this study was to examine the postoperative outcomes of eyes with advanced keratoconus that underwent femtosecond-laser zig-zag penetrating keratoplasty (z-PK) following MTK for reshaping of the central cornea. This study involved 32 eyes of 32 consecutive advanced keratoconus patients; i.e., 25 eyes of 25 patients who underwent MTK prior to z-PK (MTK + z-PK group), and 7 eyes of 7 patients who underwent z-PK alone (z-PK group). In all treated eyes, visual acuity (VA) and corneal topography were measured before surgery and at 6-months postoperative. At 6-months postoperative, the mean uncorrected distance VA (logarithm of the minimum angle of resolution) and surface regularity index (SRI) of the MTK + z-PK group was 0.62 ± 0.39 (mean ± standard deviation) and 1.26 ± 0.45, respectively, while that in the z-PK group was 1.02 ± 0.18 and 7.64 ± 3.22, respectively. Both variables were significantly better in the MTK + z-PK group than in the z-PK group (*P* < 0.05). The findings in this study reveal that MTK prior to z-PKP is effective for improving UDVA and reducing the irregularity of corneal topography in patients with advanced keratoconus.

## Introduction

Previous studies have reported that ultra-short pulse (femtosecond) laser can be used to precisely incise the transparent cornea in the eyes of patients undergoing penetrating keratoplasty (PK)^1,2^. The procedure offers a high degree of incision flexibility, and it can be applied for the creation of laser-assisted in-situ keratomileusis (LASIK) flaps^3^. Moreover, it has been reported that 'zig-zag' penetrating keratoplasty (z-PK) using femtosecond laser can produce a hermetic wound seal, as the angle edge provides a smooth transition between the host eye and the donor cornea, thus reducing residual astigmatism^4^. In addition, it is reported that the procedure results in faster recovery of vision compared to conventional techniques^5,6^.

PK is often used to treat patients afflicted with keratoconus, an eye disease that leads to thinning and deformation of the cornea^7,8^. However, in such cases, refraction abnormalities may appear post surgery. It has been reported that the average amount of astigmatism following PK is approximately 5 to 6 diopters (D)^9^. In advanced cases of keratoconus, significant astigmatism reportedly can occur even when femtosecond-laser z-PK is performed, as the host cornea is highly deformed^10^. Thus, it is necessary to correct the shape of the host cornea in such cases prior to transplantation of the corneal graft in order to reduce the risk of corneal irregularities post surgery^11^. It has been reported that one method used for correction of the corneal shape is thermokeratoplasty, a procedure that involves denaturation by heat^12,13^. Microwave thermokeratoplasty (MTK)^14^ (KERAFLEX MICROWAVE SYSTEM; Avedro, Inc. Waltham, MA) is a specialized treatment used for correction of the corneal shape in keratoconus patients, and in such cases, we perform MTK in order to correct the corneal shape prior to z-PK.

In this present study, we investigated the postoperative outcomes in eyes with advanced keratoconus that underwent femtosecond-laser z-PK following a reshaping of the central cornea by MTK.

## Methods

The protocols of this retrospective cohort study were approved by the Institutional Review Board of Kyoto Prefectural University of Medicine, Kyoto, Japan, and prior informed consent was obtained from all patients in accordance with the tenets set forth in the Declaration of Helsinki. All surgeries were performed by two surgeons (O.H., S.K.) at the Baptist Eye Institute, Kyoto, Japan between October 2008 and August 2016. The criteria for inclusion were consecutive cases of advanced keratoconus that required femtosecond-laser PK. All cases were Grade 4 keratoconus in accordance with the Amsler-Krumeich Classification for Grading Keratoconus, and deep anterior lamellar keratoplasty (DALK) was not an indication due to the high amount of corneal deformity. From 2008 to 2012, all cases underwent z-PK alone, and in 2012 and 2013, MTK + z-PK was performed only in cases with corneal protrusion not in the central region. Beginning in 2014, all advanced keratoconus cases underwent MTK + z-PK.

The MTK procedure was developed for myopia correction (up to -9D)^15^, and it involves a low energy microwave pulse being applied to the cornea at 65** °C** in order to shrink the corneal collagen fiber to the depth of 250 um. In this study, we performed MTK with a 3.8-mm inside diameter / 4.3-mm outside diameter doughnut-shape form to reduce the prominence of the keratoconus from 2-weeks to 1-month prior to the patient undergoing z-PK. The MTK procedure was performed on the most ectatic part of the host cornea, with the patient then being instructed to wear soft contact lenses for 1-week postoperative, in combination with the continuous administration of antibiotics and betamethasone eye drops until z-PK being performed. Since the effect of MTK declines rapidly post surgery^16^, z-PK was performed during the period that MTK remained effective.

**Figure 1:**
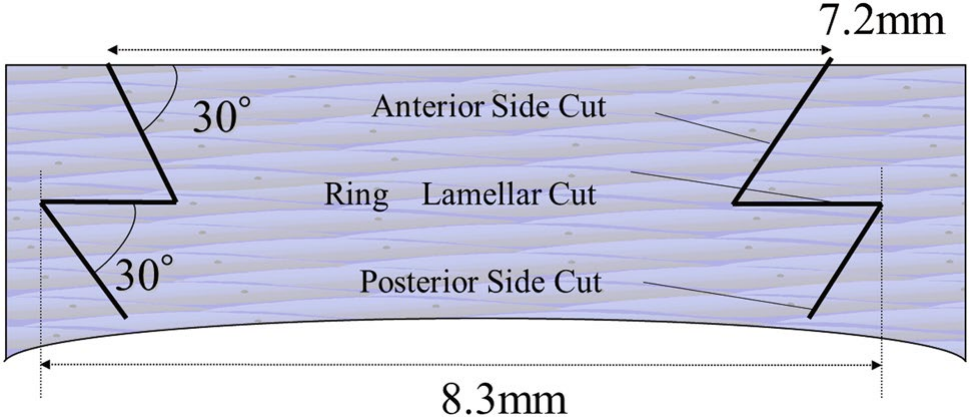
Cross-sectional diagram of the zig-zag penetrating keratoplasty (z-PK) procedure using a femtosecond laser.

In all patients, z-PK was performed with a femtosecond laser (FS60 or *i*FS; Johnson & Johnson, New Brunswick, NJ), with the corneal graft being the same size as the resection site of the host cornea (Fig. 1). The cornea was incised from the endothelium side at the angle of 30 degrees to the corneal surface. Briefly, an initial 1-mm wide horizontal incision was made at the depth of 300**-**μm from the corneal surface. Next, a 7.2-mm diameter incision was made at the corneal surface. The depth of the incision on the host eye was determined by using the average thickness from the thickness map of the anterior segment-optical coherence tomography (AS-OCT) findings and a 'half-cornea' perforation. Next, the femtosecond laser was irradiated in the laser room, with the patient then being transported to the operating room via the use of a wheelchair. z-PK was then performed under general anesthesia. The host corneal tissues left by the laser were resected using curved corneal scissors. The donor cornea with an artificial anterior chamber was resected 100% by femtosecond laser. After transplantation, the donor corneal graft was sutured 10-0 nylon interrupted sutures with 8 tissue bites, followed by 10-0 nylon running sutures with 16 tissue bites. Post surgery, the patients were prescribed antibiotics and a 4-times-daily administration of betamethasone eye drops for several years.

At 6-months postoperative, the results in all cases were compared and the running sutures were removed only in the case of loosening, and uncorrected distance visual acuity (UDVA), corrected distance VA (CDVA), and spherical equivalent refractive error (SRE) was examined. VA and SRE were measured using Landolt C charts, and VA was subjected to logarithmic transformation and analyzed as a continuous variate with logarithm of the minimum angle of resolution (logMAR).

In all treated eyes, the TMS-4 (Tomey Corporation, Nagoya, Japan) topographic modeling system was used for analysis of the corneal topography. We then evaluated the topographic astigmatism cylinder (CYL), the surface regularity index (SRI), and the surface asymmetry index (SAI), as a decrease in the SRI or SAI can contribute to CDVA improvement^17,18^.

For subgroup analysis in the MTK + z-PK group, we compared the UDVA, CDVA, SRE, CYL, SRI, and SAI between the -9D MTK + z-PK group and the -6D MTK + z-PK group.

Statistical analysis was performed using SPSS VERSION 21 STATISTICAL　ANALYSIS SOFTWARE FOR WINDOW (SPSS, Inc., Chicago, IL). Student’s t-test was used to compare the UDVA, CDVA, SRE, CYL, SRI, and SAI in the MTK + z-PK group and in the z-PK group at 6-months postoperative. A *P*-value of < 0.05 was considered statistically significant. For subgroup analysis, the variables of the -9D MTK + z-PK group and then -6D MTK + z-PK group were then compared.

## Results

**Figure 2:**
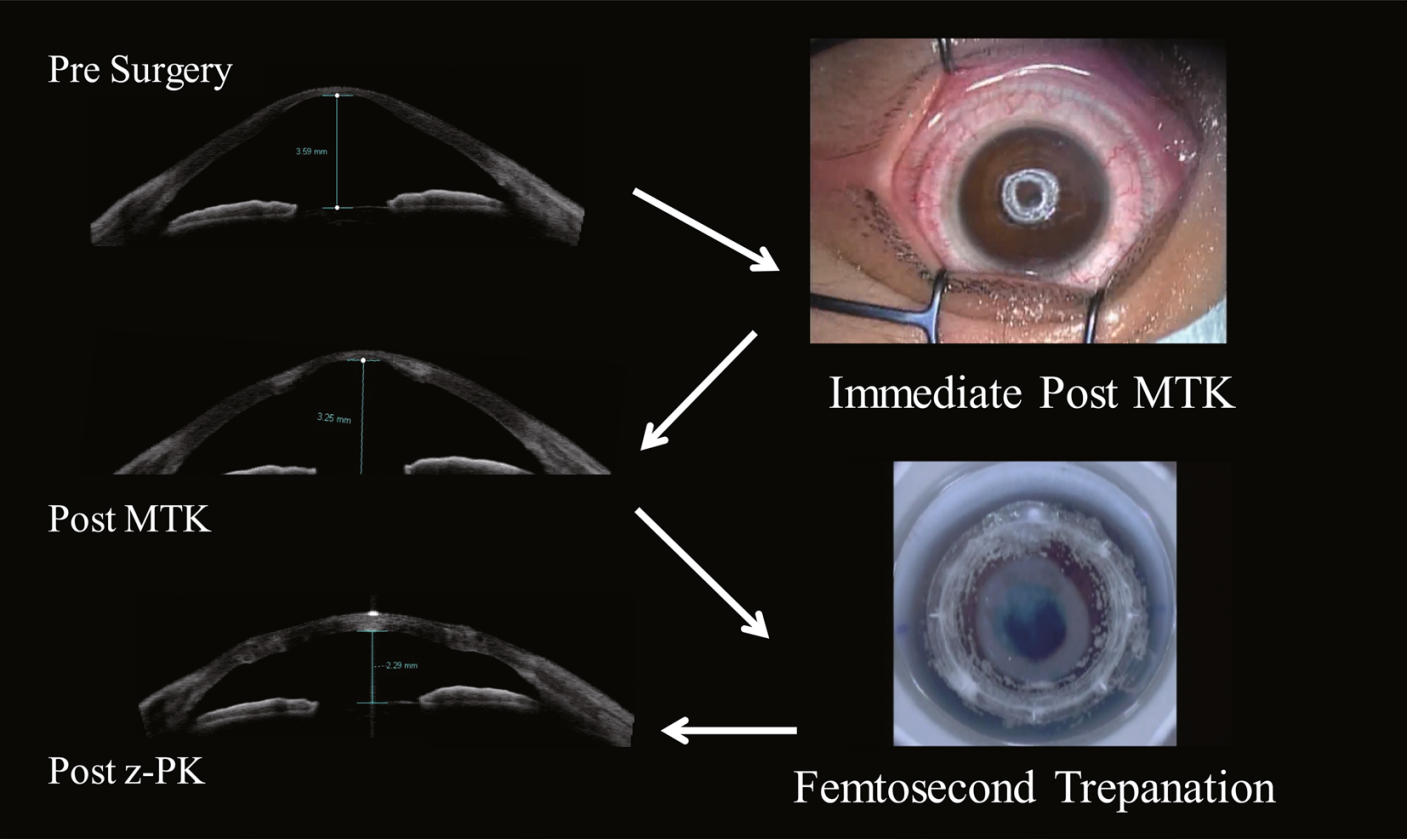
Microwave thermokeratoplasty (MTK) and z-PK images. First, MTK was performed to reduce the prominence of the keratoconus, followed by z-PK 2- to 4-weeks later.

This study involved 25 eyes of 25 consecutive patients with advanced keratoconus. In the MTK + z-PK group (Fig. 2), all patients underwent z-PK at approximately 2-weeks to 1-month post MTK. In the first 12 patients, MTK was performed mostly at the correction setting of -9D (9.2 J), while in the following 13 cases, MTK was performed at the correction setting of -6D (6.29 J). As a control to help compare the results, this study also involved 7 eyes of 7 consecutive patients with advanced keratoconus who had undergone z-PK alone (z-PK group) a few years prior to the MTK + z-PK group patients undergoing surgery.

The mean age [mean ± standard deviation (SD)] of the patients in the MTK + z-PK group and z-PK group was 43 ± 14 and 53 ± 15 years, respectively. The clinical baseline characteristics of the MTK + z-PK group and z-PK group patients are shown in Table 1. Prior to surgery, there were no statistically significant differences in age, sex, UDVA, and CDVA between the patients from both groups, yet there were 10 eyes in the MTK + z-PK group and 4 eyes in the z-PK group in which VA could not be corrected with spectacle use.

**Table 1:** Baseline characteristics of the MTK + z-PK Group and z-PK group patients.

	MTK + z-PK Group (SD)	z-PK Group (SD)	*P*-value
Patient age (years)	43 (14)	53 (15)	0.122
Males : Females	20:5	3:4	0.20
UDVA (logMAR)	1.68 (0.51)	1.74 (0.27)	0.757
CDVA (logMAR)	1.34(0.66)	1.50 (0.39)	0.534

*MTK* microwave thermokeratoplasty, *z-PK* zig-zag penetrating keratoplasty, *SD* standard deviation, *UDVA* uncorrected distance visual acuity, *logMAR* logarithm of the minimum angle of resolution, *CDVA* corrected distance visual acuity. There was no significant difference when tested with the Student's t-test.

At 6-months postoperative, the mean UDVA in the MTK + z-PK group was higher than that in the z-PK control group [i.e., 0.62 (0.39) logMAR and 1.02 (0.18) logMAR, respectively] (*P* = 0.015), the mean CDVA in the MTK + z-PK group was higher than that in the z-PK group [i.e., 0.15 (0.19) logMAR and 0.30 (0.26) logMAR, respectively] (*P* = 0.10), and the mean SRE in the MTK + z-PK group was lower than that in the z-PK group [i.e., − 2.98 (3.70) D and − 0.11 (5.37) D, respectively] (*P* = 0.10).

**Figure 3:**
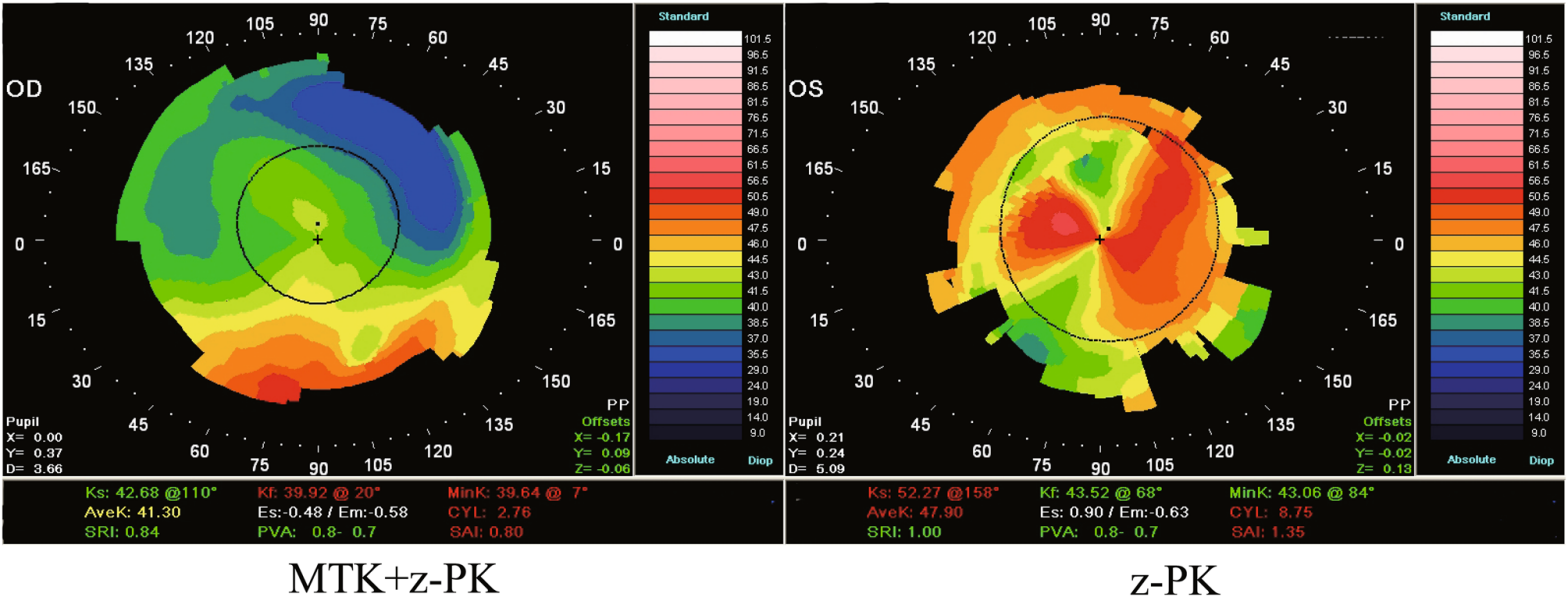
Corneal topography images of the smallest SRI in the MTK + z-PK group (left-side image) and z-PK group (right-side image). There were fewer irregularities of corneal shape in the MTK + z-PK group than in the z-PK group.

At 6-months postoperative, all patients in both groups underwent topographical analysis. The mean SRI in the MTK + z-PK group was lower than that in the z-PK group [i.e., 1.26 (0.45) and 1.63 (0.32), respectively] (*P* = 0.045). The corneal topography of the eye with the smallest SRI in each group is shown in Fig. 3. There was also a lower average of astigmatism and SAI in the MTK + z-PK group (*P* = 0.24 and *P* = 0.53, respectively). The postoperative clinical variables are shown in Table 2.

**Table 2:** Comparison of the postoperative outcomes between the MTK + z-PK group and z-PK group at 6-months postoperative.

	MTK + z-PK Group (SD)	z-PK Group (SD)	*P*-value
UDVA (logMAR)	0.62 (0.39)	1.02 (0.18)	0.02*
CDVA (logMAR)	0.15 (0.19)	0.30 (0.26)	0.10
SRE (D)	− 2.98 (3.70)	− 0.11 (5.37)	0.11
CYL (D)	6.24 (2.59)	7.64 (3.22)	0.24
SRI	1.26 (0.45)	1.63 (0.32)	0.05*
SAI	1.24 (0.68)	1.62 (1.21)	0.53

*MTK* microwave thermokeratoplasty, *z-PK* zig-zag penetrating keratoplasty, *SD* standard deviation, *UDVA* uncorrected distance visual acuity, *logMAR* logarithm of the minimum angle of resolution, *CDVA* corrected distance visual acuity, *SRE* spherical equivalent refractive error, *D* diopters, *CYL* topographic astigmatism cylinder, *SRI* surface regularity index, *SAI* surface asymmetry index.

*There was a significant difference when tested with the Student's t-test.

During the study period, no complications leading to visual impairment were observed in both groups. However, 3 patients in the MTK + z-PK group and 2 patients in the z-PK group underwent z-PK and simultaneous cataract surgery, with 4 of those 5 cases undergoing ultrasound cataract surgery in open sky with intraocular lens fixed in the capsular bag; 1 eye in the z-PK group requiring intraocular lens suturing due to ciliary zonule weakness. The mean elapsed period between MTK and z-PK in the MTK + z-PK group was 24 ± 16 days (range: 7–90 days). In 2 of the 12 MTK + z-PK group eyes that underwent MTK at the -9D setting (9.2 J), the procedure resulted in severe inflammation and pain. Thus, additional systemic and topical administration of steroids was required for 1-week following the procedure.

Subgroup analysis revealed that there was no significant difference in the preoperative and postoperative variables of the –9D MTK + z-PK group and the -6D MTK + z-PK group (supplement 1). However, the -9D MTK + z-PK group did include 3 patients that underwent z-PK and simultaneous cataract surgery.

## Discussion

Our findings revealed that in cases of advanced keratoconus, MTK performed before femtosecond-laser z-PK leads to significant improvement of UDVA and SRI at 6-months postoperative.

Busin et al. previously reported that corneal cauterization performed prior to transplantation of the corneal graft resulted in a decrease of refractive error and astigmatism^11^. However, no corneal topography index measurements were performed in that study. The data in this present study indicates that thermokeratoplasty can make a CDVA-correlated significant improvement of the corneal topography index post z-PK. Thus, thermokeratoplasty via the microwave system may create a smoother corneal surface after PK than corneal cauterization performed manually.

In this study, the use of MTK led to the reduction of average astigmatism. The average astigmatism was more than 6D may be due to the fact that all cases were advanced keratoconus. In Japan, when the keratoconus is within the moderate level, ophthalmologists generally prescribe the use of hard contact lenses instead of corneal transplantation. In this present study, all patients underwent the same suture technique and suture management method. However, it has been reported that depending on the differences between specific suture management methods applied, it may be possible to reduce astigmatism to a greater extend^19^.

In the subgroup analysis, no significant differences in postoperative UDVA, CDVA, CYL, SRI, and SAI were observed between the -9D MTK (9.2 J) + z-PK group and the -6D MTK (6.92 J) + z-PK group. The -9D MTK + z-PK group included 2 cases of severe inflammation post MTK, so we currently perform MTK via the standard MTK method with the setting of -6D correction.

It should be noted that this study had some limitations. First, only a small number of cases were involved in the 6-month follow-up. Second, the study included cases that underwent simultaneous cataract surgery, which might have had an influence on the refractive errors. Our findings revealed that performing MTK prior to PK leads to improvement of UDVA at 6-months postoperative, and at our institution, treated keratoconus patients are allowed to start wearing hard contact lenses at 6-months after PK. In the future, we plan to further investigate the effects of MTK + z-PK by extending the follow-up period and including cases with trephine PK only.

In conclusion, our findings revealed that performing MTK prior to femtosecond-laser z-PK was effective for the improvement of UDVA and corneal shape in cases of advanced keratoconus, even when a femtosecond laser is used for surgery.

## Supplementary Information

Supplementary Information
