# Supplementary material for: Comparison of penetrating keratoplasty outcomes with or without microwave thermokeratoplasty
Source: Sci Rep. 2021 Mar 16;11:5995. doi: 10.1038/s41598-021-85470-5 (PMC7966373; doi:10.1038/s41598-021-85470-5)
Supplement: Supplementary file 1 — Supplementary Information [file 41598_2021_85470_MOESM1_ESM.docx]

**Supplement 1 Table.** Comparison between the -9D MTK + z-PKP Group and -6D MTK + z-PKP Group at 6-Months Postoperative (Subgroup Analysis)

|  | -9D MTK + z-PKP Group (SD) | -6D MTK + z-PKP Group  (SD) | *P*-value |
| --- | --- | --- | --- |
| Patient age (years) 43 (14) 53 (15) 0.122  Males : Females 20:5 3:4 0.20  Pre-UDVA (logMAR) | 44(16)  4 : 9  1.68 (0.64) | 42(13)  1 : 11  1.68 (0.38) | 0.63  0.32  0.45 |
| Pre-CDVA (logMAR)  Post-UDVA (logMAR)  Post-CDVA (logMAR)  Post-SRE (D) | 1.30(0.77)  0.57(0.39)  0.17(0.19)  -1.79(3.66)  1.40(0.5 1.40(0.58) | 1.40(0.58)  0.68(0.21)  0.14(0.21)  -4.08(3.51)  1.40(0.58) 1.40(0.58) | 0.75  0.48  0.70  0.13 |
| CYL (D) | 6.29(2.00) | 6.17(3.20) | 0.92 |
| SRI | 1.36 (0.51) | 1.14 (0.35) | 0.22 |
| SAI | 1.37 (0.80) | 1.23(0.58) | 0.62 |

D = diopters; MTK = microwave thermokeratoplasty; z-PKP = zig-zag penetrating keratoplasty; SD = standard deviation; UDVA = uncorrected distance visual acuity; logMAR = logarithm of the minimum angle of resolution; CDVA = corrected distance visual acuity; SRE = spherical equivalent refractive error; CYL = topographic astigmatism cylinder; SRI = surface regularity index; SAI = surface asymmetry index.

______________________________________________________________________
